# A Protest against Quackery*This paper was prepared for the Forty-First Annual Meeting of the V. D. A., but was not reached for want of time.—Ed. Reg.

**Published:** 1885-05

**Authors:** G. D. Billings

**Affiliations:** Medina, O.


### COMMUNICATIONS.

### A Protest Against Quackery.*

BY G. D. BILLINGS, MEDINA, O.

*Gentlemen of the Mississippi Valley Dental Society:*

Being requested by a member of your Executive Committee to be present with you at your Forty-First Annual Meeting or send a short paper; as I cannot be with you, and as subordination to civil, social, scientific, and other laws is a virtue, I therefore write. As my thoughts at this time (and of late years quite frequently) are in the channel of one branch of our profession, and as it seems to me the backward in place of forward steps in

*This paper was prepared for the Forty-First Annual Meeting of the M. V. D. A., but was not reached for want of time.—Ed. Reg.

what I consider a noble and high calling. *Dental science*—if those words mean anything they mean a great deal—I cannot help thinking of our old and honored members, in our noble State of Ohio, Drs. Watt, Taft, Keely, Berry, and many others who have worked so hard for nearly a life time; in fact some of them for more than a half century, until their locks have been silvered with grey, until they are white as snow. All those years they have worked faithfully and well, never tiring of the determination to work for the good of their chosen calling; to elevate and educate those younger in life's battles and trials; to see the art of dentistry elevated to a high position. To see it, or at least a portion of it, dropping and still going lower and lower, until to-day it is not on a level with many of our so-called trades, is indeed discouraging to them; how they must feel! God pity them, for I fear that I do not know how.

The portion or part to which I allude is prosthetic, or artificial dentistry.

Gentlemen, I feel that there is a just and noble God or ruler of this universe, and if it be true, there must be a very large book, full to overflowing with sentences of greater or lesser degrees, for those who call themselves dentists, (not only in my neighborhood, but in close proximity to each and every one of you,) for the deformed condition of his chosen people, caused by their desire and determination to grasp the almighty dollar, regardless of future consequences. This they do by removing many natural teeth that could be saved for years to come, and putting in their places cheap, *ungodly*, ghastly-looking *things*, at prices ranging from $5 to $7 per plate, of fourteen things, attached thereto. I say things, yes, I repeat it, *things!* God forbid that I should so far forget myself as to call them by anything but their proper names.

Contemplate the following scene, if you please. A beautiful painting by one of our finest artists, a life-size portrait of a man upon canvas. This painting was by the artist proclaimed to be a perfect picture of General Geo. H. Thomas; but hold! the General is tall, broad shouldered, full chest, the legs symmetrical, and the whole form, indeed, is all that could be wished. There is one exception that alone has ruined the picture. The arms are just twelve inches from the shoulder to the tip or end of the index finger. This, when looked upon by one of the lowest, most ignorant, degraded sots, that our beloved country is cursed with, though he had not smiled for many years, would cause him to burst into such a peal of laughter as to attract the attention of all those around about him and cause them to look upon the deformed representation upon canvas, of our good and departed General—Pap Thomas. But still there are met every day by each of us upon the streets, people much more deformed than the sad mistake the artist made of the General, and yet we pass them by without raising our voices in holy horror at the sight. In a late number of a dental journal I read that cheap artificial teeth were a good thing. Now, I am compelled upon general principles to take issue with the writer, for I cannot consistently see where the good thing comes in any more with that kind of work than with any other. For example: I have within my twenty years of practice seen many cases of so-called artificial teeth that were not worth carrying home, the hard earned money which was paid for them was worse than thrown away. Within the last eighteen months, two superior plates have come into my office, one of them had shed every one of the so-called teeth, whilst three, or one block, was remaining on the other. Neither of those plates had been in use for one year. These are only specimens of the so-called cheap artificial dentistry, yet those noble men making this grade of deformity told the poor deluded souls that all the difference there was in articial teeth was in the price that different dentists charged for them. Now what is to be done about it, says one. I humbly acknowledge my inability to answer, but am willing to advance a few thoughts which, if carried out, to my mind, would in time bring about at least a partial reformation, viz: That every dentist belonging to or hereafter joining a society of dentists for the advancement of the art be required to sign a by-law, so worded as to kill forever, this continual backbiting and jealousy, and put a stop to derogatory remarks about our honest competitors. We ought to do all within our power to bring about kindly feeling for each other, or in other words, for each and every one of us, to take upon himself the role (as near as it is possible for us poor mortals to do) of the guardian angel, and do all within our power to elevate not only our competitors, but the people in the community wherein we may be located. We should compel, by kindly words and acts, our neighbor co-workers to become friends. It would be worse than useless for one alone to try to do this. All of the good honest members of the profession united, what a power they would have! Will you do it? I wish to give a little of my own experience in this connection, then I am through: Eighteen years ago I landed at the place I have since made my home, and have tried to do honestly by my patrons. I was a stranger, but in a few hours I purchased the outfit and good will of a dentist. After I took possession of the office, he took great pains to warn me against having anything to do with a brother dentist who was in business, saying that if I did it would ruin my prospects whilst on earth, and those for the future. I will not try to enumerate all the mean things that were placed before me about him. I was young, and, all in all, it was a trying place for me, a stranger in a strange land. I had been taught by my honored preceptor to treat all dentists as men and be willing at all times to look over the short comings of those that I felt had not treated me right, for they were only men, like other people. I stood between two fires, one told me if I did, I would be d——d, the other that if I did the other thing I would *surely* be. I was compelled after a couple of days to follow the instructions of my *old* friend and instructor. (The last remark may cause him to rise from his chair, as if a pin was there, as he does not appreciate being called old.) I called upon this demon dentist, as he was represented to be, and lo! and behold, I found a man, yes a gentleman, and I continued to call upon him, and in time he returned the compliment. We have lived all these years side by side, and never had a harsh word or thought to my knowledge, and I feel to-day that I have not a better friend, or one who would do me a favor quicker, than this very same dentist. Brothers, can't you go and do likewise?

